# Environmental genomics of "*Haloquadratum walsbyi*" in a saltern crystallizer indicates a large pool of accessory genes in an otherwise coherent species

**DOI:** 10.1186/1471-2164-7-171

**Published:** 2006-07-04

**Authors:** Boris A Legault, Arantxa Lopez-Lopez, Jose Carlos Alba-Casado, W Ford Doolittle, Henk Bolhuis, Francisco Rodriguez-Valera, R Thane Papke

**Affiliations:** 1 Evolutionary Genomics Group, División de Microbiología, Universidad Miguel Hernández, Apartado 18, San Juan 03550, Alicante, Spain; 2 Canadian Institute for Advanced Research, Centre Robert Cedergren, Departement de Biochimie, Universite de Montreal, 2900 Boulevard Edouard-Montpetit, Montreal, Quebec, H3T 1J4, Canada; 3 Canadian Institute for Advanced Research Program in Evolutionary Biology, Department of Biochemistry and Molecular Biology Dalhousie University, Halifax, Nova Scotia, Canada; 4 Department of Microbial Ecology, Centre of Ecological and Evolutionary Studies, University of Groningen, Kerklaan 30, 9751 NN Haren, The Netherlands; 5 Genome Atlantic and Department of Biochemistry and Molecular Biology Dalhousie University, Halifax, Nova Scotia, Canada

## Abstract

**Background:**

Mature saturated brine (crystallizers) communities are largely dominated (>80% of cells) by the square halophilic archaeon "*Haloquadratum walsbyi*". The recent cultivation of the strain HBSQ001 and thesequencing of its genome allows comparison with the metagenome of this taxonomically simplified environment. Similar studies carried out in other extreme environments have revealed very little diversity in gene content among the cell lineages present.

**Results:**

The metagenome of the microbial community of a crystallizer pond has been analyzed by end sequencing a 2000 clone fosmid library and comparing the sequences obtained with the genome sequence of "*Haloquadratum walsbyi*". The genome of the sequenced strain was retrieved nearly complete within this environmental DNA library. However, many ORF's that could be ascribed to the "*Haloquadratum*" metapopulation by common genome characteristics or scaffolding to the strain genome were not present in the specific sequenced isolate. Particularly, three regions of the sequenced genome were associated with multiple rearrangements and the presence of different genes from the metapopulation. Many transposition and phage related genes were found within this pool which, together with the associated atypical GC content in these areas, supports lateral gene transfer mediated by these elements as the most probable genetic cause of this variability. Additionally, these sequences were highly enriched in putative regulatory and signal transduction functions.

**Conclusion:**

These results point to a large pan-genome (total gene repertoire of the genus/species) even in this highly specialized extremophile and at a single geographic location. The extensive gene repertoire is what might be expected of a population that exploits a diverse nutrient pool, resulting from the degradation of biomass produced at lower salinities.

## Background

Extreme environments provide simplified microbial communities which may be usefully compared and contrasted with more complex ecosystems. Community complexity is often assessed in terms of the diversity of phylogenetic markers such as 16S ribosomal RNA – but generally we do not know the extent to which organisms with identical or very similar sequences for phylogenetic sequences differ in other shared genes and in gene content. Indeed, complete genome sequences for multiple strains of the same species (usually cultivatable pathogenic organisms) often reveal widely different gene complements [1-4]. Genes present in only a few strains of a species have been described as "accessory or adaptive", since they do not appear to be essential for cell survival, but instead provide the possibility to adapt to environmental fluctuations (for example, use different nutrients) [5]. Knowledge about accessory gene pools (their sizes and compositions) will be essential in areas from ecology (how many different phenotypes can be encompassed by cells carrying this marker) to biotechnology (gene diversity available for exploitation).

One approach to assessing a species' or accessory gene pool is to compare a sequenced genome to a metagenomic sequence database obtained from an environment in which that organism dominates. In this way the usual biases and limitations associated with comparing different isolates from the same species by complete genome sequencing and/or substractive or microarray hybridization are avoided. A recent paper describing an acid mine drainage biofilm metagenome using shotgun cloning and sequencing detected very little gene content diversity among two dominant species in the community [6]. Our results suggest a different pattern for saltern crystallizers.

Saltern crystallizers (NaCl saturated) have been studied extensively by molecular methods and prove to be among the simplest known communities in terms of species composition by both classical microbiological and molecular methods [7-12]. The saturated NaCl concentrations together with high levels of magnesium inhibit the growth of all but the most resilient hyperhalophilic archaea and bacteria [13]. A number of molecular approaches have shown that the dense prokaryotic populations (reaching up to 10^8 ^cells per ml) are largely dominated by members of the recently cultivated square-shaped archaeon "*Haloquadratum walsbyi*" [7-9,11,14]. This organism was first described in 1980 before the recognition of the domain *Archaea *and was for a long time known as "Walsby's square bacterium" [15]. The species appears cosmopolitan: 16S rDNA sequences retrieved from these haloarchaea do not diverge by more than 1.6% worldwide,]: Spanish and Australian isolates [16] differ only in two nucleotides across the whole 16S rRNA gene [17]. The peculiar morphology of this organism (square flattened cells of 2–5 μm sides) permits enrichment by filtration ; smaller rod and spiral-shaped cells pass through a 2 μm pore size filter and the square-shaped cells are retained. The genome of the Spanish isolate HQSB001 has been fully sequenced and annotated and we had access to the annotated genome of this strain [18].

In this work we isolated biomass from a saltern crystallizer with the aim of retrieving mostly "*H. walsbyi*"- related material, and compared the recovered gene diversity with that of the sequenced strain's genome, isolated specifically from this same pond two years earlier [17]. The results of this study, which involved end-sequencing of ca. 2000 fosmid clones suggest that "*H. walsbyi*" shares a large accessory gene pool that could easily exceed the size of the sequenced strain's genome.

## Results

### Metagenomic library coverage and taxonomic diversity

The crystallizer pond populations from which this DNA was extracted has been the focus of a series of molecular studies over the last 12 years, and we have substantial information regarding the prokaryotic composition of this particular pond [7,8,10-12,14,19-21]. However, the microbial community could be affected by long term or seasonal changes so we used PCR amplification, cloning and sequencing to characterize the specific sample used for metagenomic library construction and as a control for the suitability of the filtration method used for sample fractionation (see materials and methods). We sequenced about 40 cloned 16S rDNA genes from each fraction (2 μm and 0.2 μm). As expected the proportion of "*Haloquadratum*"-related clones was appreciably higher in the 2 μm fraction (66% of the total of the clones) than the 0.2 μm fraction (50%). Additionally, *Salinibacter ruber*, the other major component of the community [22] was effectively removed from the 2 μm fraction (2% of the total clones in the 2 μm filter versus 15% on the 0.2 μm filter) which is confirmed in the fosmid library analysis (see below). As expected, the diversity of both *Archaea *and *Bacteria *was very restricted. "*Haloquadratum*"-related clones were predominant in the archaeal primers clone library. Out of 40 clones 27 were highly similar to "*Haloquadratum*" seven were similar to *Haloarchaeon *CSW2.24.4 [GenBank: AY498650], an isolate from a crystallizer pond in Victoria, Australia [23], three were similar to *Halosimplex carlsbadense *and three were similar to *Halorubrum tibetense*. Among the "*Haloquadratum*"-related clones the variability was extremely low, with most sequences diverging less than 1%. Moreover these clones were highly related (≥ 98.8%) to sequences retrieved from the same pond by PCR amplification twelve years ago [7]. Overall, the "*Haloquadratum*" affiliated 16S rDNA gene sequences retrieved from this pond in different surveys and with different methodologies were highly conserved, and never diverged more than 1.6%, reflecting the high degree of homogeneity of the 16S rRNA genes of this species and/or the stability of the population in this crystallizer.

**Figure 1:**
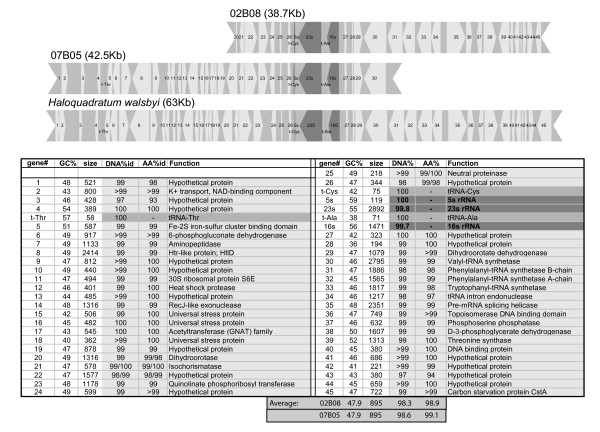
**Environmental fosmids 02B08 and 07B05 and comparison with corresponding region on the "*Haloquadratum walsbyi*" HBSQ001 chromosome**. The table underneath displays identity and similarities of each gene from the environmental clones compared to the genome sequence. In the overlapping areas two values are shown corresponding the gene in 02B08 and 07B05 respectively. When only one value is shown both similarities differed only by decimals.

The fosmid library constructed from the 2 μm fraction was composed of ca 2000 clones with an average insert size of 35 Kb. Thus, the total amount of cloned environmental DNA is in the order of 70 Mb. Given that the genomic diversity of this environment was previously calculated to be to 7 *E. coli *genome equivalents (i.e. 30 Mb) by DNA/DNA reassociation [24] the size of the library was considered sufficient to cover the numerically dominant "*Haloquadratum*" related lineages. PCR screening of the metagenome library revealed eight 16S rRNA genes, five of which were highly related to "*Haloquadratum walsbyi*" (4 were 100% identical and one had six nucleotide differences in a 500 bp fragment). Of the remaining three, two were related to *Haloarchaeon *CSW4.24.4 [GenBank: AY498650] and one to the genus *Halosimplex*. Fosmids that contained one of the rRNA operons of "*Haloquadratum*" (02B08 and 07B05), were fully sequenced and compared with the "*H. walsbyi*" strain HBSQ001 genome [25]. The similarity is very high throughout this fragment indicating how highly conserved this part of the genome is (Figure 1). Strain HBSQ001 was isolated two years before the samples used in this study were obtained. Every year the crystallizer ponds are emptied to harvest the salt and then refilled with water from lower salinity ponds. Therefore the environmental DNA and the strain do not come from the same body of water although the location is the same.

#### Analysis of the library by fosmid end-sequencing

**Figure 2:**
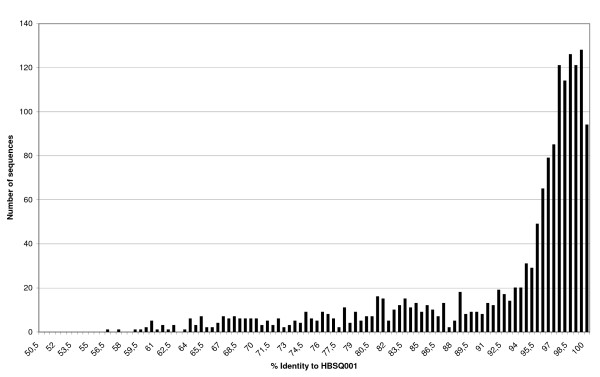
Histogram distribution of DNA sequence identity to HBSQ001 among fosmid-end sequences.

**Figure 3:**
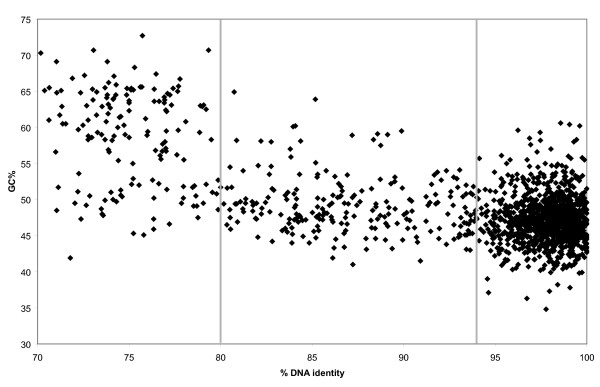
**Scatter plot of DNA sequence identity to HBSQ001 versus GC-content**. Each dot corresponds to one fosmid-end sequence.

**Figure 4:**
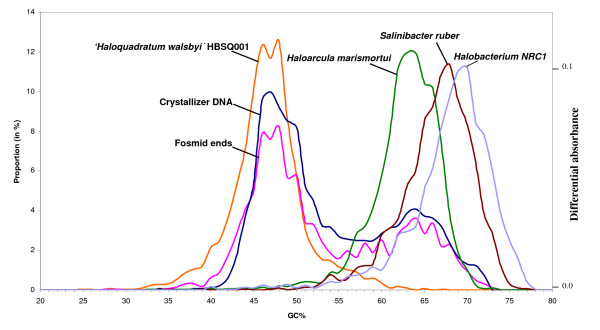
**GC% distribution of genes from selected halophilic genomes, of fosmid-end sequences and of crystallizer DNA**. GC values of individual genomes were obtained from public databases except for *Salinibacter ruber *and "*Haloquadratum walsbyi*" that were obtained from their respective genome databases [42, 43]. The GC plot from the crystallizer DNA determined by reassociation kinetics (differential absorbance) is from reference [12].

For this work fosmids ends were sequenced using vector primers. We regularly obtained sequences over 500 nucleotides and the success rate was acceptable (~74%). In total 2948 individual end sequences were determined with an average size of 818 bps, generating ca 2.4 Mb of DNA sequence data. Given the availability of the complete "*H. walsbyi*" strain HBSQ001 genome sequence [25], comparison of the metagenome library was straightforward (Figure 2). One thousand and twenty-nine sequences (1029) with more than 94% nucleotide identity to the HBSQ001 genome were considered as bona-fide "*H. walsbyi*" (belonging to lineages closely related to the sequenced strain). This range of sequence similarity is commonly observed with groups designated as "species" by other criteria [26]. Another set of 261 sequences with 80% to 94% identity to the HBSQ001 genome was categorized as "*Haloquadratum*". Finally, 173 sequences with no similarity to the HBSQ001 genome on one end of the fosmid insert, but in which the other end showed >80% DNA identity with HBSQ001 were given the "*Haloquadratum*"-scaffold (Hq-scaffold) tag. The majority of these 173 sequences have a low GC content, which is characteristic of genes in the sequenced HBSQ001 genome and which is unlike all other known haloarchaeal and hyperhalophilic bacterial genomes [12]. The fosmids depicted in Figure 3 and indeed of most sequences in the metagenome library (Figure 4) have this unique and defining characteristic.

**Figure 5:**
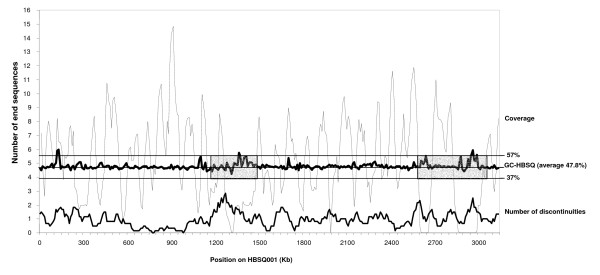
**Distribution of the fosmid-end sequences along the HBSQ001 chromosome showing coverage and discontinuities**. Position for each fosmid-end is obtained from the best blast hit between the fosmid-end and the HBSQ001 chromosome. 'GC-HBSQ001' consists of the number of guanine (G) and cytosine (C) nucleotides out of every 10 nucleotide on the HBSQ001 chromosome (averaged over 10 Kb). At any corresponding nucleotide position on the HBSQ001 genome (x-axis): 'coverage' consists in the number (averaged over 10 Kb) of fosmids from the library where both ends have more than 80% DNA identity to HBSQ001 and for which distance between corresponding BLAST hit positions on the HBSQ001 genome is smaller than 60 Kb; discontinuities consist of the number (averaged over 60 Kb) of 1) fosmid end sequences with more than 80% DNA identity to HBSQ001 but for which the other end gives no hit to HBSQ001 and 2) fosmids for which distance between corresponding BLAST hit positions on the HBSQ001 chromosome is greater than 60 Kb. "Choppy" regions are highlited by a grey background.

Most of these fosmid inserts had sequences at both ends with high similarity to HBSQ001. Assuming that fosmid inserts are shorter in length than 60 Kb and may correspond to identical regions on the HBSQ001 genome, synteny between the cloned environmental DNA fragments and HBSQ001 could be assessed. We identified 457 fosmids in which both ends had hits located at less than 60 Kb apart (average 35.2 Kb) and with >80% DNA identity (average 96.4%). Concatenation of the sequences from these syntenic fosmid inserts would have allowed the reconstruction of more than 92% of the HBSQ001 chromosome, with an average coverage of 5.7X (Figure 5). This analysis also identified 66 fosmid inserts in which both ends have at least 80% DNA identity to HBSQ001 but which are more than 60 Kb apart on the HSBQ001 genome. Indeed, the average distance between the corresponding HBSQ001 sequences was 589 Kb, indicating that large genomic rearrangements had taken place in the HBSQ001 genome or the genome from which the fosmid insert came. Rearrangments seemed to correlate somewhat with DNA divergence since 24% (30 out of 123) of the fosmid inserts with at least one end having between 80% and 94% identity with the strain HBSQ001 were rearranged, compared with only 9% (37 out of 402) of those in which both ends had >94% DNA identity.

The 173 fosmids having similarity to HBSQ001 at just one end must represent borderline sections in the genomes of the metapopulation where synteny with the sequenced strain is broken. The location of these sequences, plus the sequences from the 66 fosmid inserts in which there is greater than 60 Kb between HBSQ001 homologs were plotted onto the HBSQ001 genome (Figure 5). Results from this analysis indicate that the distribution of these discontinuities is widespread in the genome, but is by no means random and there may be rearrangement "hot-spots". A GC content plot for the HBSQ001 genome shows two relatively "choppy" regions (large high and low peaks) each about 0.5 Mb interspersed with relatively "calm" regions with fewer and smaller peaks. The frequency of the discontinuity events is correlated with larger amplitude variance in the GC content (Figure 5). In particular, a large segment of 541 Kb starting at position 543 Kb, contains only 11 discontinuities (on average, one per 49.2 Kb) in spite of being heavily covered in the metagenome library, whereas the contiguous 324 Kb region, within the first choppy region, contains 37 discontinuities (one per 8.5 Kb). A lack of synteny is also associated with the low level of HBSQ genome coverage from the environmental library (Figure 5). This observation is consistent with these regions being present in the sequenced strain but not in many of the environmental lineages. The accessory gene pools of pathogenic bacteria are also found in regions of atypical GC content [27].

**Figure 6:**
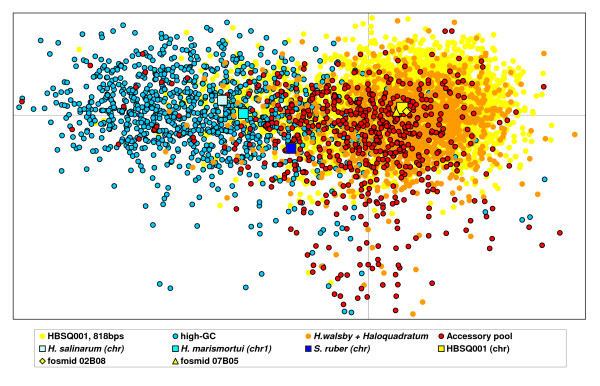
**Trinucleotide analysis of fosmid end sequences**. The distance between any two dots (sequences) represent relative proximity between their sequence signatures (multidimensional scaling was performed from 64-dimensions to 2-dimensions). The genome of HBSQ001, cut in chunks of 818 bp (the average size of fosmid-end sequences) is represented by dots tagged "HBSQ001, 818 bp". Dots with the "*H. walsbyi*" + "*Haloquadratum*", "high-GC" and "accessory pool" tags represent fosmid ends sequences from corresponding categories as described in the text.

Taking advantage of the fact that HBSQ001 has an average GC-content that is nearly 20% lower than all other known hyperhalophiles, we also identified metagenomic sequences as coming from "*Haloquadratum*" by analyzing their GC-content (Figure 3 and 4) [28]. Establishing an upper cut-off value of 55%, allowed categorization of 414 sequences with no similarity to the HBSQ001 genome (which has a GC-content lower than 55%) as most likely belonging to "*Haloquadratum*" lineages. Trinucleotide analysis (Figure 6) shows that a clear majority of these "low-GC" sequences possess a "*H. waslsbyi*"- like sequence signature, supporting our hypothesis that most of these indeed consist of sequences found associated with cells within the "*Haloquadratum*" population. These 414 sequences together with the 173 sequences previously identified as Hq-scaffold must represent the accessory pool for this species in this specific environment since they are not present in the sequenced strain and therefore cannot be essential for its survival and contained only within certain lineages of the environmental diversity of the species. Hereafter we will refer to this combined set as the "accessory sequence pool".

It is also noteworthy that we found 17 fosmids with high similarity (at least at one end to the *Salinibacter ruber *genome. The only other organism to which similarities over 94% nucleotide identity to protein coding genes were found.

### Functional analysis of the accessory gene pool

As expected, a majority of fosmid-insert ends with high DNA sequence identity to the HBSQ001 genome (~1200) also had a best amino-acid BLAST hit to putative proteins in HBSQ001. These sequences represent a bit more than 28% of its genome and have an average amino acid identity greater than 91%. Most interesting are the sets of end-sequences lacking DNA similarity to HBSQ001 but in the "accessory sequence pool". Among the 587 such sequences, 232 (40%) had best hits to putative "*H. walsbyi*" (average aa. identity of 61%) or other haloarchaeal genes, 51 (9%) were similar to other archaea or bacteria, and another 304 (52%) had no hit in public databases. One hundred and twenty-four of these 304 sequences had predicted ORFs of 100 amino-acids long or longer. A low theoretical pI value, which is characteristic of halophilic proteomes [29] was found for a majority of these predicted ORFs, indicating that at least some of them may be translated into functional proteins.

**Figure 7:**
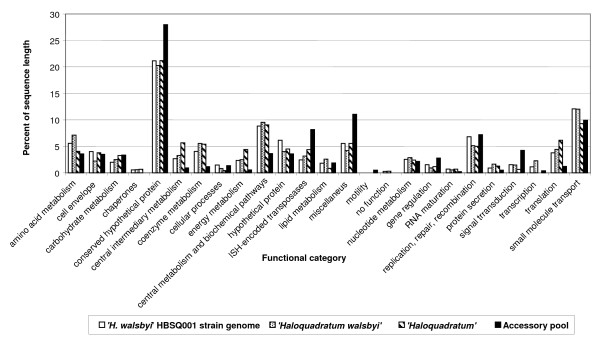
**Functional category distribution of putative genes found on fosmid-end sequences and comparison with "*H. walsbyi*" HBSQ001 genes**. "*H. walsbyi*", "*Haloquadratum*" and "Accessory pool" are all putative genes found in the corresponding sequence categories described in the text.

Functional category distribution analysis of the 283 accessory gene pool sequences, strain HBSQ001, "*H. walsbyi*" fosmid-ends, and "*Haloquadratum*" fosmid-ends, revealed important differences in several categories (Figure 7). Indeed, some "core" functional categories are clearly underrepresented in the "accessory sequence pool", such as that of transcription, translation, and energy metabolism. Conversely "peripheral" functional categories such as signal transduction and gene regulation are overrepresented in that set, as well as IS-encoded transposases, conserved hypothetical proteins and sequences with ill-defined function (miscellaneous). Unexpectedly a relatively large number of cell-envelope components were also detected in this gene pool.

## Discussion

During the last few years there has been a dramatic change in the understanding of the dynamics of microbial genomes. The tradition established by the first population genetics studies carried out in the 1980's was to consider that mutation was the driving factor in bacterial adaptation and diversification, as is the case in model metazoan and plant organisms. However, comparative genomics has changed this view, indicating that indeed the main variable that allows adaptation to different niches is the presence of variable gene pools in different strains. These so-called accessory pools are involved in differences in pathogenecity and many other properties that are strain-specific [29,30]. Most of the information obtained in this field derives from comparing different isolates from the same designated species. The problem with using single isolates is that there is always a certain bias of isolation and it is difficult to infer how representative such isolates are of their natural populations. This is particularly an issue when dealing with non pathogenic organisms with which there may also be considerable microdiversity in the sample or habitat [3,31]. So far, the most thorough attempt to retrieve information from directly sequencing an environmental DNA dominated by one species indicated very little variability in gene content among the lineages present in it [6] although a considerable sexual exchange was detected among lineages within the population.

Our study hints to a potentially large gene reservoir in the saltern habitat. The roughly 1/3 of sequences retrieved with high GC values and no similarity to either "*Haloquadratum*" or *Salinibacter *(or to any GenBank entry) belong with all probability to other haloarcheal (or bacterial) species present in the crystallizer that had not been detected previously by 16S rDNA amplification and sequencing, T-RFLP or FISH and demonstrate the limitations of these techniques. Still approximately 2/3 of the fosmid library most likely belong to "*H. walsbyi*" or closely related lineages, as shown by the similarity of the 16S rRNA genes retrieved from the library, the percent GC and trinucleotide frequency values and, in many cases, the high similarity to the sequenced strain HBSQ001. In this sense it is remarkable to observe that nearly identical sequences were found for the two completely sequenced fosmid inserts that contained rRNA ribosomal operons. This, together with the virtual retrieval of the near complete HBSQ001 genome, hints to the permanence of a "*H. walsbyi*" backbone genome with a high degree of conservation. But it is also apparent from this work that there are other lineages sharing the environment that differ in (i) synteny (ii) gene content and probably also (iii) pool of paralogous genes. Some "choppy" clusters rich in transposable elements found in the HBSQ001 strain appear to be underrepresented in the population captured in the fosmid library. On the other hand, many genes present in the library also appear to be absent from the sequenced "*H. walsbyi*" isolate. The architecture of the metagenome would be as depicted in Figure 5. The fraction of genes (about 80% of a typical strain genome size) that is shared by most (or all) the lineages would represent the "core" genome [27] of the species. This core contains the genes that provide essential cell biology functions and in which variation would be deleterious (see below). On the other hand, certain regions at relatively conserved locations of individual genomes contain highly variable sets of genes, which we identify as "accessory". Although such genes represent a minority for any specific individual genome (about 20%) the total number of genes contained within this variable compartment could be very large (in the present case not less than 3 Mb or a genome equivalent). The large fluctuations with respect to the average genome GC content (together with the study of the corresponding regions of the HBSQ001 genome) suggest an involvement of IS elements and phage related genes in the the evolutionary dynamics of this accessory gene pool.

The salterrn crystallizer studied is barely 20 cm deep and no benthic communities are known. Still, this environment could offer a variety of microniches. Suspended particules could harbor different microbiota, with different nutrional characteristics, as has been demonstated for open-ocean environments [32,33]. Diel and seasonal cycles might generate ecological diversity, and the complexity of the nutrient pool in the crystallizers is great. The major nutrient source would be dead and lysed cells of the eukaryotic alga *Dunaliella*, which is the primary producer at lower salinities. Full utilization of this complex resource would require either an enormous genome encoding all required metabolic pathways or a diverse pool of lineages each with slightly different degradative properties.

Our data suggest that there is indeed a diverse pool of lineages. The enrichment in signal transduction (environmental sensing) and gene regulation in the accessory pool (that is among genes found only in some strains) suggests a key role in microadaptation to slightly different niches. Transcriptional regulators or signal transduction could produce diversity in the response to environmental change that might optimize resource exploitation under varying conditions. This is particularly remarkable for a non motile organism like "*Haloquadratum*". The production of gas-vesicles must provide some control over cell location in their habitat, but the responses to environmental gradients must be rather limited. Less expected but also present among accessory genes were those encoding cell envelope components which might be involved in phage evasion (there are no other predators in the crystallizer). Suggestive as these correlations are, we cannot at the moment discount the possibility that many of the differences in gene content are effectively neutral, and only observable because of a high rate of DNA exchange [3].

Our results contribute to an appreciation of the problems associated with assembling complete genome of any single uncultured strain in such a complex community of related strains, for example when shotgun strategies are used [34,35]. Conserved genes can be identified and conserved regions might be mapped, but lack of synteny and variable gene content in other regions would preclude the scaffolding of any complete genome. Thus complete sequencing of the genomes of selected isolates in pure culture remains a valuable and possibly essential adjunct to metagenomic studies such as ours.

## Conclusion

Nearly all of the genes in the genome of the sequenced strain "*Haloquadratum walsbyi*" (HBSQ001) were recovered from our environmental DNA library, and many of our fosmids exhibited synteny with that genome. HQSB001 was isolated from the same saltern as our environmental DNA samples but two years before.

The pan-genome of this metapopulation in this specific habitat and location contains at least another genome equivalent (ca 3 Mb) in addition to the genome of the sequenced strain

The differential gene content is found associated mostly with regions of atypical GC content and a high frequency of phage/IS elements, suggesting an involvement of such elements and horizontal gene transfer in the maintenance of the accessory gene pool. The accessory genes often appear to be involved in regulation and signal transduction, underscoring the importance of these processes in the adaptation of specific lineages to specific heterotrophic microniches in this nutrient rich habitat.

## Methods

### Sampling, DNA extraction and 16S rRNA gene PCR amplification, clone libraries construction and sequencing

The sample used to construct the genomic and the clone libraries was collected from the crystallizer pond CR30 in the multi-pond saltern "Bras del Port" located in Santa Pola (Alicante, Spain, 38° 12' N, 0° 36' W) in November 2002. The salinity was measured *in situ *with a hand refractometer (Atago). The sample was prefiltered through a filter paper to remove the biomass of small eucaryotic organisms (e.g. mosquitoes and copepods) and other particles in suspension. Then it was left still at room temperature for two hours in order to recover the green alga *Dunaliella salina*, very abundant in the environment at the time of the sampling, from the surface of the bottle by aspiration. The sample was then filtered sequentially through 2 μm (Isopore, Millipore) and 0.2 μm pore size filters Durapore, Millipore). Filters were stored at -80°C until DNA extraction. Thawed filters were cut with a sterile razor blade in small pieces and vortexed in 2 ml of sterile water in 15 ml conical base polypropylene centrifuge tubes. The supernatant was transferred to a new tube, and 0.1 vol of 10% sodium dodecil sulphate and proteinase K (0.5 mg/ml) were added. The tubes were incubated at 55°C for 2 h and in a boiling water bath for 2 min. The lysates were extracted twice with phenol-clhoroform-isoamyl alcohol (25:24:1) and once with chloroform-isoamyl alcohol (24:1). The purified nucleic acids were concentrated by ethanol precipitation, resuspended in sterile Milli-Q water and stored at -20°C.

PCR 16S rRNA gene amplification was carried out using the 16S rDNA archaeal-specific primer 21F (TTCCGGTTGATCCTGCCGGA) and 16S rDNA bacterial-specific primer ANT1 (AGAGTTTGATCATGGCTCAG) and using the universal primer S, as reverse (GGTTACCTTGTTACGACTT) for both. Two independent PCR reactions for each primer combination (*Archaea *and *Bacteria *domain) were carried out under the following standard conditions: 35 cycles (denaturation at 94°C for 15 s, annealing at 55°C for 30 s, extension at 72°C for 3 min) preceded by 2 min denaturation at 94°C and followed by 5 min extension at 72°C. PCR products obtained of each primer combination were purified with Qiaquick PCR purification kit (Qiagen). Clone libraries were constructed using the Topo TA cloning system (Invitrogen) according to manufacturer recommendations. The plasmids of positives clones were extracted and measured with Hoescht fluorescent dye. The concentration of the plasmid/PCR product was adjusted to 0.075 pM in 6 μl by dilution with sterile water. The sequencing reaction was performed by PCR amplification in a final volume of 10 μl using 0.05 pM of plasmid, 4 pmoles of primer, and 1 to 3 μl of Big Dye Terminators premix, according to Applied Biosystems protocol. After heating to 98°C for 5 min, the reaction was cycled as follows: 30–40 cycles of 30s at 96°C, 30s at 55°C, and 4 min at 60°C (9700 thermal cycler AB). Removal of excess of Big Dye Terminators was performed by ethanol precipitation. The samples were dried in a vacuum centrifuge, dissolved in 10 μl of formamide, loaded onto an Applied Biosystems 3730 sequencer and run for 2 hours. For sequencing, primers B1055 (for bacterial clones) and Arc915 (for archaeal clones) were used.

### Genomic library construction

Our environmental genomic library was constructed with DNA corresponding to the >2 μm biomass fraction. We started from an initial amount of ~3.5 mg of DNA, as estimated by comparison with different amounts of linear T7 DNA in agarose gels. DNA was broken by mechanical shearing and fractionated in a 0.8% low-melting point agarose gel after DNA ends were repaired (EpiFOS™ Fosmid Library Production Kit, Epicentre). The band corresponding to 35–45 Kb DNA fragments was cut from the gel, digested with gelase and cloned in the 7500 bp-EpiFOS™-5 Fosmid Vector (Epicentre). The ligated fosmids were packaged in phage particles and used to transfect *E. coli *EPI100™ (Epicentre). A total of 2000 fosmid clones were obtained in this way, i.e. an initial library of 70–80 Mb, assuming an average insert size of 35–40 Kb.

### End sequencing of fosmids clones

The DNA from fosmid clones was prepared using the Eppendorf Perfectprep-96 BAC DNA Extraction Kit (Brinkmann) and the quality of DNA was checked on a 1% agarose gel after a NotI digestion. The sequencing reactions were performed using approximately 500 ng of template with half-strength ET Terminator reactions (Amersham Biosciences) in both forward and reverse directions. The reaction products were cleaned and analyzed using a MegaBACE 1000 DNA Sequencer (Amersham Biosciences). Primers used: T3 and T7 (AAT TAA CCC TCA CTA AAG GG and GTA ATA CGA CTCACT ATA GGG C respectively) (Integrated DNA Technologies).

### Metagenomic library screening and sequencing of fosmid clones containing 16S rRNA genes

The library was pooled in groups of 48 clones (50 μl of each clone) that served for PCR screening. The fosmids were extracted with Qiaprep Spin Miniprep kit (Qiagen) and resuspended in 50 μl of sterile Milli-Q water. The PCR screening and 16S rRNA gene sequence was carried out as above. Eight pools gave expected-size (~1.5 Kb) amplification products. Further PCR screening of individual clones integrating these pools led to the identification of eight archaeal 16S rDNA-containing fosmid clones. The eight amplicons were fully sequenced using primers 21F, 640R (GGATTTCACTCCTACCCC), 958R (ACCGGCGTTGACTCCAATT), and 1492R. From these, only clones 02B08 and 07B05 affiliated to "*Haloquadratum walsbyi*", were fully sequenced (Genome Express), DNA was purified from a 1 ml overnight culture. A shotgun library was generated by subcloning mechanically sheared DNA (2.5–5 Kb) into pUC18. Inserts were sequenced with vector primers using a MegaBase 4000 capillary sequencer (Amershan Biosciences).

#### Fosmid end sequences DNA-analysis

The BLAST program blastn [36] was used to compare the end sequences against the complete genome sequence of "*Haloquadratum walsbyi*" [25]. The following blast parameters were used: "-q -1 -r 1 -F F". All sequences with a blast hit to HBSQ001 of at least 100 base pairs and more than 94% DNA-DNA identity were categorized as "*H. walsbyi*", >94%" while hits to HBSQ001 of at least 100 bps and between 80% and 94% DNA-DNA identity were classified as "*Haloquadratum*". These cutoffs of 80% and 94% were chosen based on the distribution of the blast hits (Figures 2, 3). For some fosmids, a significant (>100 bps, >80% identity) hit was found only at one end. In those cases, the other end sequence, although it did not show DNA similarity to the genome sequence of "*H. walsbyi*", was assigned to belong to a set of "*Haloquadratum" *-scaffold sequences. Finally each of the remaining sequences were binned as either "low-GC" (GC-content less or equal to 55%) or "high-GC" (GC-content greater than 55%), based on the analysis of GC-content distribution of end sequences, which clearly showed two sets with nearly no overlap, one peaking around 48%, the average GC-value of "*Haloquadratum*", and another less sharp peak around 64%, close to the average GC-value of most other known hyperhalophiles. Several perl scripts were written to investigate the distribution of hits along the "*H. walsbyi*" chromosome. A perl program was also written to perfom trinucleotide analysis, based on the formula described in [37]. Multidimensional scaling of trinucleotide signatures to 2-dimensions was performed using the software RuG/L04 [38].  Fosmid end sequences have been deposited in GenBank with accession numbers  DU824018 to DU826964.

#### Fosmid end sequences functional analysis

The program BLAST blastx (translated DNA vs protein) was used to extract functional information from end sequences. All end sequences were blasted against the nr database and against "*Haloquadratum*". All hits below 30 amino acids or with an e-value greater than 10e-5 were considered non-significant, and all hits with a score below 80 or with a best hit to eukaryotes were manually verified. For each sequence where a significant hit could be found, another round of BLAST was performed on parts of the sequence not covered by the best blast hit. Preliminary analysis of the results of this additional gene fragments showed that they did not impact the results significantly and so they were ignored in the final results compilation. Functional categories for ORFs with a best match in HBSQ001 were assigned based on the HBSQ001 genome annotation [25]. The functional category was assigned manually for all other hits, using the HBSQ001 categories as a template. For end sequences without hit to any known sequence, the large ORFs (>100 a.a) were identified using getorf program from EMBOSS package [39]. Isoelectric point calculations were made using the Sequence manipulating suite web interface [40].

#### Analysis and annotation of fosmids 02B08 and 07B05

Potential ORFs for the two complete fosmids 02B08 (38.7 Kb) and 07B05 (42.5Kb) were extracted using the gene finding program glimmer [41]. Spacers were subsequently blasted against the nr public database to ensure that no ORF had been missed. The HBSQ001 genome annotation was also helpful in confirming potential ORFs, start positions and annotation for the two fosmids. DNA similarity comparisons between the two fosmids and with HBSQ001 was performed using the BLAST program blastn, with parameters "-q -1 -r 1 -F F".

## Authors' contributions

BAL carried out annotation and analysis of the fosmid-ends sequences (DNA and functional analyses and comparisons), annotation and comparative analysis of fosmids 02B08 and 07B05, and participated in the writing of the manuscript, mostly the results section. AL-L collected and filtered saltern samples and made the fosmid library, screening for the presence of 16S rRNA genes and the sequencing of those fosmids. JCA constructed the 16S rDNA clone libraries and made the phylogenetic analyses of the sequences retrieved. BAL and JCA made the figures. FR-V, WFD, HB, and RTP conceived the work. FRV and WFD wrote the manuscript. RTP collected and filtered saltern samples, sequenced fosmid ends and contributed bioinformatic analyses. All the authors read and approved the final manuscript.
